# Therapeutic Effect of Exosomes Derived From Stem Cells in Spinal Cord Injury: A Systematic Review Based on Animal Studies

**DOI:** 10.3389/fneur.2022.847444

**Published:** 2022-03-10

**Authors:** Cangyu Zhang, Rongrong Deng, Guangzhi Zhang, Xuegang He, Haiwei Chen, Bao Chen, Lin Wan, Xuewen Kang

**Affiliations:** ^1^Department of Orthopaedics, The Second Hospital of Lanzhou University, Lanzhou, China; ^2^Key Laboratory of Osteoarthritis of Gansu Province, Lanzhou, China; ^3^Department of Nephrology, The Second Hospital of Lanzhou University, Lanzhou, China

**Keywords:** stem cell, exosomes, spinal cord injury, animal study, systematic review

## Abstract

**Objective:**

A systematic review of the role of stem cell-derived exosomes in repairing spinal cord injury (SCI) and the existing problems in animal experiments to provide a reference for better animal experiments and clinical studies in the future.

**Method:**

Three electronic databases, namely PubMed, Web of Science, and Ovid-Embase were searched. The studies were retrieved from inception to October 2021. Two researchers independently screened the literature, extracted data, and evaluated the methodological quality based on the inclusion criteria.

**Results and Discussion:**

Thirty-two studies were incorporated into the final analyses. Exosomes derived from stem cells could not only significantly improve the motor function of animals with SCI, but also significantly increase the expression of anti-inflammatory factors IL-4 and IL-10 and anti-apoptotic protein Bcl-2, while significantly lowering the pro-inflammatory factor IL-1β and TNF-α and the expression of the apoptotic protein BAX. However, the mechanism of exosome-mediated SCI repair, as well as the best source and dosage remain unknown. In addition, there are still some issues with the design, implementation, and reporting of animal experiments in the included studies. Therefore, future research should further standardize the implementation and reporting of animal studies and fully explore the best strategies for exosomes to repair SCI so as to promote the translation of preclinical research results to clinical research better and faster.

## Introduction

Spinal cord injury (SCI) is a devastating disease that can result in impaired sensorimotor function, defects in the autonomic nervous system, and neuropathic pain (1). The annual incidence of SCI is as high as 15–40 cases per 100,000, and the global mortality rate of hospitalized patients with acute SCI is as high as 4.4–16.7, which is on the rise worldwide (2). While SCI causes severe pain for patients, it also has a significant financial impact. Although various treatments such as drugs, physical therapy, hyperbaric oxygen therapy, and surgical intervention have been used clinically, the complicated pathological conditions of SCI, for instance extensive cell death and severe inflammation make these treatments difficult to obtain satisfactory results (3). The greatest challenge at the moment is promoting nerve function recovery after SCI.

Stem cell therapy has shown promise in the treatment of SCI in recent years, which can effectively promote axon regeneration, inhibit astrocyte scar, and reduce inflammation (4). Although the efficacy of stem cells in the treatment of SCI has been confirmed in animal studies, the results of animal experiments have not been easily translated into clinical practice. The main reasons are the low survival rate of stem cell transplantation, limited targeting ability, difficulty in passing through the blood-brain barrier, prone to immune rejection, dedifferentiation, formation of malignant tumor formation, etc. (5, 6). In addition, some studies have revealed that the transplantation efficiency of stem cells is extremely low, where only 1% of the transplanted stem cells can migrate to the target tissue. Most of the stem cells transplanted by vein gather in the lung or liver, and cannot reach the SCI site to play a repair role (7, 8). Further studies have shown that the restoration of nerve function of damaged spinal cord induced by stem cells depends on the transmission of intercellular signals through paracrine and endocrine mechanisms, rather than the regeneration of transplanted stem cells. Among them, exosomes, as the main substances secreted by stem cells, may play a major role in the treatment of SCI and are considered to be the best potential choice (9, 10).

Exosomes are extracellular vesicles with a diameter between 30 and 100 nm that are actively secreted by cells. They are an important medium for material transfer and information exchange between cells. They can cross the blood-brain barrier and transfer proteins, lipids, DNA, and RNA transfers to target cells and regulate their biological processes (11). Compared with transplanted stem cells, exosomes have the advantages of long residence time *in vivo*, low carcinogenicity, high targeting efficiency, low immune rejection, easy to pass through the blood-brain barrier, and easy to obtain and store (12–15). In addition, the sources of exosomes are diverse and can be derived from various cells. In particular, exosomes derived from stem cells can mimic the phenotype of parental stem cells, thereby activating the self-renewal program of target cells to achieve the purpose of treating diseases (16). Several studies have shown that exosomes derived from mesenchymal stem cells can significantly reduce neuronal apoptosis and inflammation, promote the regeneration of blood vessels in the injured spinal cord and, thus, promote the functional recovery of the injured spinal cord (17–19). However, the application of exosomes in SCI still has certain controversies and limitations. For example, Huang et al. tracked exosomes through an animal imaging system and found that most of the exosomes still gather in the liver and lungs, and are finally swallowed by monocytes. In fact, only a limited number of exosomes reach the spinal cord (20, 21). However, Chen et al. reported that exosomes are much smaller than stem cells and are easily accessible through the blood-brain barrier without being captured by liver or lung tissue (22).

Although exosomes derived from stem cells have made important progress in the repair of SCI in animal experiments, the current unclear and inconsistent research results have limited the further study of exosome therapy to a certain extent and it also hinders the pace of clinical translation of exosome therapy. Therefore, as the first systematic review in this field, we intend to comprehensively evaluate whether and to what extent stem cell-derived exosomes can repair SCI and the problems existing in current animal experiments by strictly evaluating the risk of bias, quality of evidence, internal and external authenticity of animal experiments, with a view to better promoting the development of animal experiments and the translation of animal experimental results to clinical studies in the future.

## Materials and Methods

### Inclusion and Exclusion Criteria

#### Subjects

Animal models of SCI were included, without limiting animal species and modeling methods.

#### Interventions

The exosomes are derived from stem cells. Stem cell-derived extracellular vesicles were excluded because extracellular vesicles are vesicles that fall off the membrane or are secreted from cells, ranging in diameter from 40 to 1,000 nm, contain a large number of proteins, mRNA and miRNA, etc. These components also have a function to regulate macrophage phenotype and inhibit the inflammatory response and have not been well-studied (23). Extracellular vesicles can be further divided into three main types according to their release mechanism and size: exosomes (<150 nm in diameter), microvesicles, and apoptotic bodies (both larger than 100 nm) (24). Exosomes are the only ones with the smallest diameter.

#### Comparisons: Normal Saline, PBS, and Other Negative Controls

##### Outcomes

1) **Basso–Beattie–Bresnahan (BBB) locomotor rating scale**

The BBB score ranges from 0 to 21, which can directly reflect the recovery of motor function. The higher the score, the better the recovery of the rat's motor function (25).

2) **Apoptotic Protein and Anti-Apoptotic Protein**

The expression levels of BAX and Bcl-2 protein in animals with SCI were measured by Western blot analysis.

3) **Pro-Inflammatory Factors**

The expression levels of IL-1β and TNF-α in animals with SCI were measured by Elisa.

4) **Anti-Inflammatory Factor**

The expression levels of IL-4 and IL-10 in animals with SCI were measured by Elisa.

#### Type of Study

Control studies were included, with no restrictions on the blind method.

#### Exclusion Criteria

Repeatedly published studies; non-Chinese and English literature; no access to full text and/or studies with incomplete data.

### Search Strategy

We searched scientific databases such as PubMed, Ovid-Embase, and Web of Science. The relevant literature was retrieved from inception to October 2021. The search terms were (exosome OR exosomes OR secretome OR allochthon OR exosomal) AND (spinal cord compression OR spinal cord contusions OR SCI OR spinal cord injuries OR spinal injuries OR spinal cord trauma OR spinal cord transection OR spinal cord laceration OR post traumatic myelopathy), refer to Supplementary Table 1 for full search strategies of each database.

### Literature Screening and Data Extraction

Two trained researchers selected the articles and stringently extracted the data based on the inclusion/exclusion criteria, and the selections were cross-checked. In the case of disagreement, a third researcher settled the conflict with a common consensus. Data were extracted according to the pre-established full-text data extraction checklist, including (1) Basic characteristics of studies such as authors, publication years, country, type of study; sex, age, body weight, sample size, and modeling methods of the animals; type, source, dose, route, timing of transplantation of exosomes; and intervention measures in the control group. (2) Outcomes: BBB score, apoptotic protein and anti-apoptotic protein, pro-inflammatory factors, and anti-inflammatory factor.

### Risk of Bias Assessment

Based on SYRCLE's risk of bias tool for animal studies (26), two trained researchers independently evaluated and crosschecked the inherent risk of bias in the included studies, covering selection bias, implementation bias, measurement bias, follow-up bias, report bias, and other bias from a list of 10 questions or tools. A difference in opinions were negotiated or decided by a third party. The answer to the assessment questions (tools) should be either “yes” that indicated low-risk of bias, or “no” that indicated high-risk of bias. For unclear items, an answer with “unclear” was assigned.

### Quality Assessment of the Evidence

Whether the results of a systematic review of animal studies can lead to clinical translation depends on the quality of the evidence. Based on the GRADE evidence grading system (27), quantitative indicators were used to assess the evidence quality in the following five aspects: (1) research limitation; (2) inconsistency of results; (3) indirectness; (4) inaccuracy; and (5) publication bias. First of all, the evidence quality of each result was evaluated, and then the evaluation results of each part were integrated to achieve the grades of evidence: high, medium, low, and extremely low.

### Statistical Analysis

STATA 16 software was used for statistical analysis. Weighted mean difference (WMD) was used to study the effect analysis statistic for continuous variable and the 95% CI as the effect amount. Heterogeneity of results between studies was assessed by a χ^2^-test (the significant level for the heterogeneity test was *p* = 0.1). Also, *I*^2^ was used to judge the degree of heterogeneity. The fixed-effect model was used for meta-analysis if the research results were not statistically different. Conversely, if there were statistical heterogeneity, the source of heterogeneity was further analyzed, and the random-effects model was used for meta-analysis after the exclusion of evident clinical heterogeneity. The significance level (*p*-value) for tests was set at 0.05. A funnel chart was drawn for the publication bias test.

## Results

**Figure 1:**
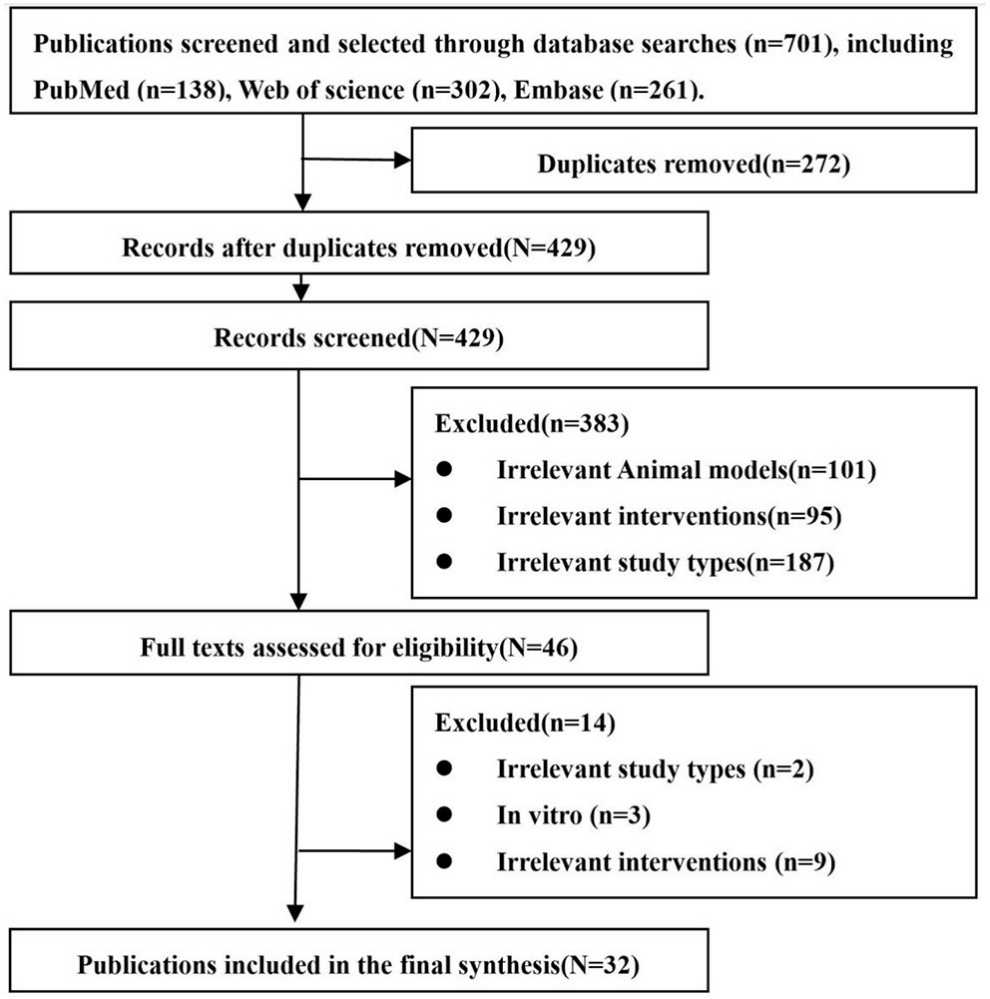
Flow chart of literature screening.

A total of 701 related articles were obtained. After excluding the literature based on the exclusion criteria, eventually 32 studies were included (19–21, 28–56). The entire screening process is summarized in Figure 1.

### Basic Information for Inclusion in the Study

Among the included 32 studies, only two studies were controlled studies (29, 33), and the remaining 30 studies were randomized controlled studies. The species of animals included SD rats (19–21, 28–37, 39, 40, 42–44, 46, 47, 49–53, 55, 56), Wistar rats (41, 54), and C57BL/6 mice (38, 45, 48); gender included male (28, 30–32, 38–45, 49, 53, 54) and female (19, 20, 29, 33, 34, 36, 37, 46–48, 50–52, 55, 56), two studies did not report the sex of the animals (21, 35); the weights of animals were between 150 (41) and 300 g (20, 44), the weight of the mouse was between 17 and 22 g (48), and seven of the studies did not report the weight of the animals (21, 29, 30, 38, 42, 45, 50); age was between 6 (38, 44, 45, 50, 51) and 12 weeks (47, 56), and nine of the studies did not report the age of the animals (19, 21, 35–37, 42, 46, 51, 52); samples size were between 10 (51) and 100 (41); models of SCI included contusion (20, 21, 28–32, 34–53, 56), hemisection (54), and transection (19, 33, 55); the types of exosomes included the exosomes of bone marrow mesenchymal stem cells of SD rats (BMSCs-Exo) (20, 21, 28, 30, 34–37, 39, 42–44, 47, 49, 52, 53, 56), BMSCs-Exo of Wistar rats (41, 54), BMSCs-Exo of human (19, 33, 50), exosomes of human placental mesenchymal stem cells (HpMSC-Exo) (55), exosomes of human umbilical cord mesenchymal stem cells (hUC-MSC-Exo) (48, 51), and exosomes of neural stem cells from SD rats (NSCs-Exo) (29). Six studies reported types of exosomes but did not report specific sources (31, 32, 38, 40, 45, 46). The transplantation route of exosomes included tail vein (19–21, 29–32, 34–50, 52–56), intranasally (33) and subcutaneous (51), and one study did not report the route of exosome transplantation (28); the time transplantation of exosome ranged from 0 (19, 20, 31, 38, 41, 44–47, 49) to 24 h (42, 43) after modeling, one study did not report the time of exosome transplantation (40); dose of exosomes ranged from 20 (29) to 200 μg (28, 30–33, 36–38, 41, 43, 45–49, 53, 56); the control group includes PBS (19–21, 28–32, 34, 35, 38, 39, 41, 43–51, 53–56), saline (33, 36, 37, 40, 42), and blank (52). Basic information of the study subjects was summarized in Supplementary Table 2.

### Results From Assessing the Risk of Bias, Quality of Evidence, and Publication Bias

**Figure 2:**
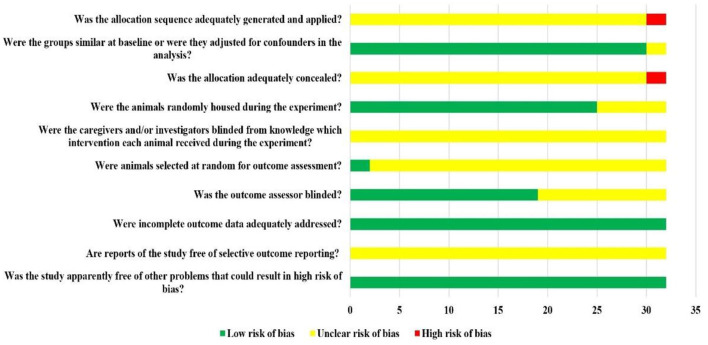
Risk of bias of each item of SYRCLE tool for overall included studies. Each risk of bias item presented as percentages across all included studies, which indicated the proportion of the different level risk of bias for each item.

Among the included 32 studies, 30 studies were randomized controlled studies, but they did not report the specific randomization method and whether covert groupings are implemented. A total of 30 studies reported the similarity of baseline characteristics such as age, sex, or weight of animals; in total, 25 studies reported randomized placement of animals during the experiment. Based on the limited information provided by the included studies, none of the studies were able to determine whether or not blinding animal breeders and/or researchers. Only two studies reported measuring results by randomly selecting animals, only 19 studies blinded the evaluators of the results. All the experimental animals of 32 studies were included in the final analysis, although no research protocol was available for any of the studies, all expected results were reported. The risk of bias assessment for all studies is detailed in Figure 2.

Among the seven outcome indicators, the quality of evidence was either “low” or “very low.” The reasons for the degradation of evidence quality included the lack of intrinsic authenticity of animal studies and the possibility of large publication bias. The results of the evidence quality assessment included in the study are shown in Supplementary Table 3.

**Figure 3:**
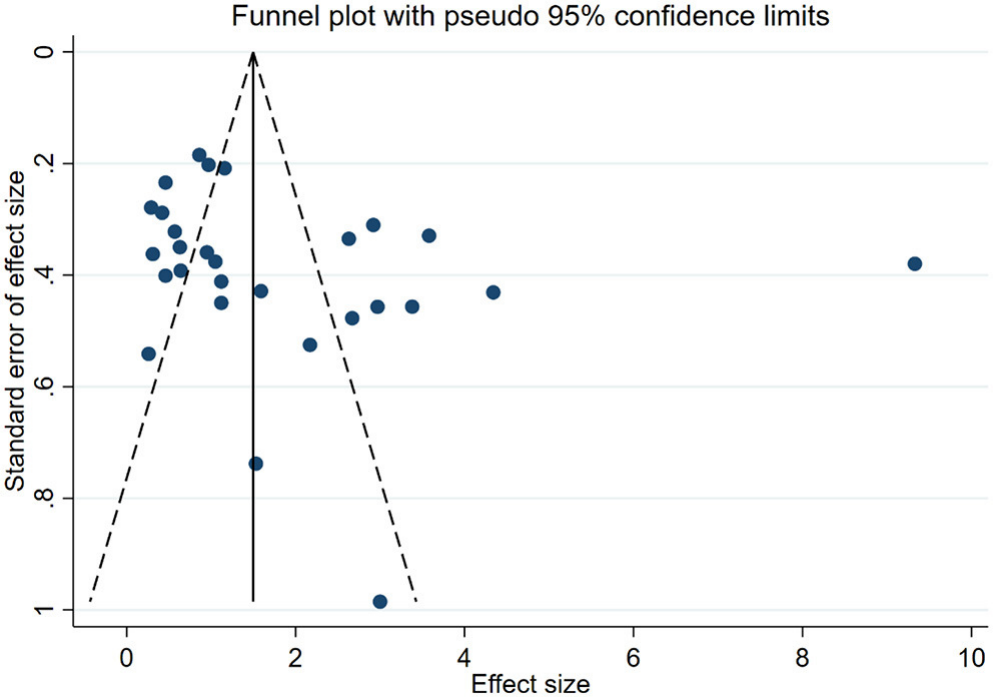
Funnel plot of BBB score at the 1st week.

Through the publication bias detection of the research data of 1 week of BBB score, the results showed that the funnel plot was asymmetric, suggesting that there was a certain publication bias in the included research (Figure 3).

### Results of the Meta-Analysis

#### BBB Score

**Figure 4:**
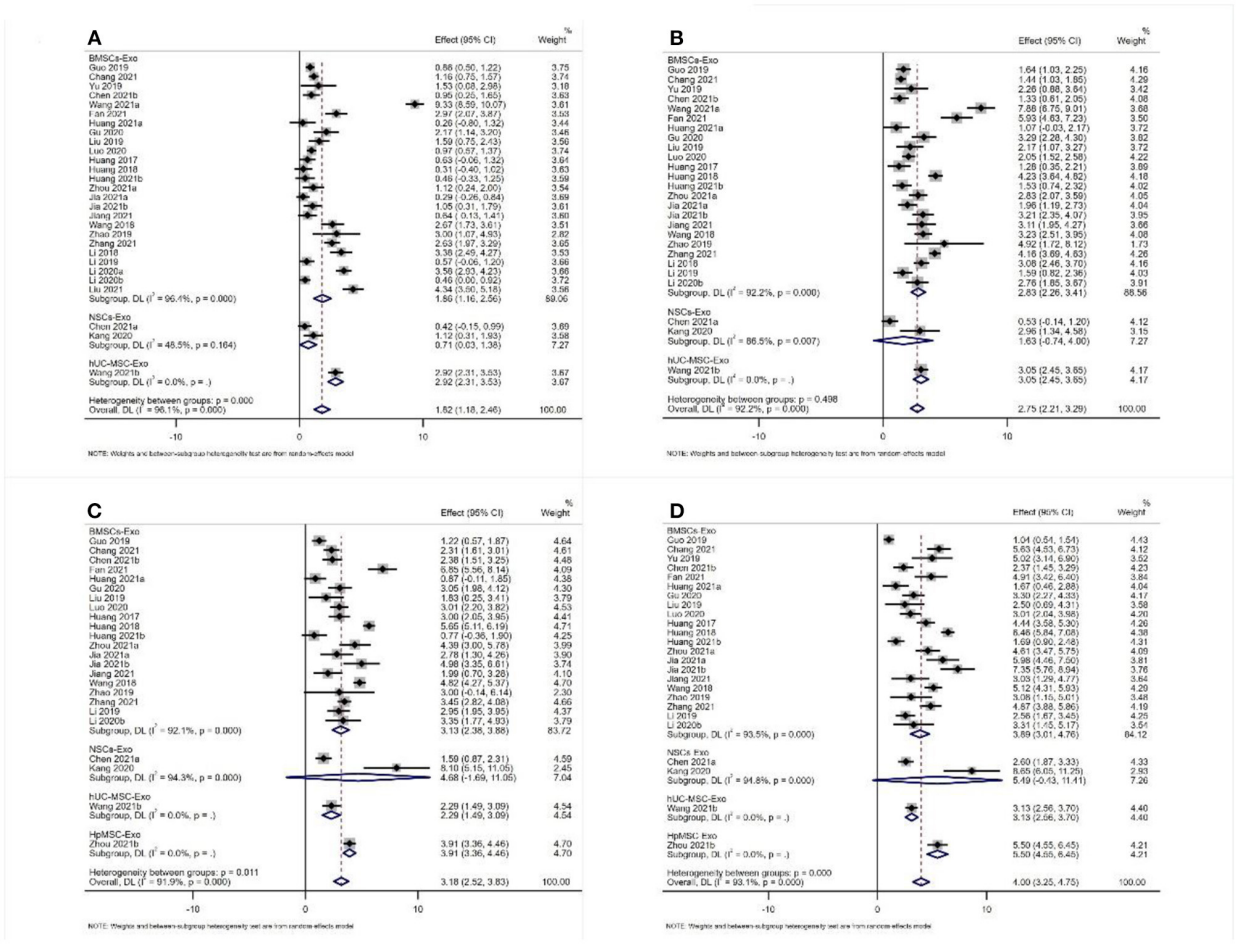
Meta-analysis results of BBB score **(A)** 1st week; **(B)** 2nd week; **(C)** 3rd week; **(D)** 4th week.

Twenty-nine studies reported the results of BBB scores of animals after exosome transplantation (19–21, 28–37, 39–44, 46, 47, 49–56). The meta-analysis results of the random effects model showed that the BBB score of the exosome group was significantly better than that of the negative control group, and the difference was statistically significant [1st week after transplantation: WMD = 1.82 (1.18, 2.46). Second week after transplantation: WMD = 2.75 (2.21, 2.39), 3rd week after transplantation: WMD = 3.18 (2.52, 3.83), and 4th week after transplantation: WMD = 4.00 (3.25, 4.75), Figure 4]. The abovementioned meta-analysis results are a combined analysis of the research results of different animals, modeling methods, different sources, transplantation routes, and transplantation doses of exosomes. Although the results of different periods are very consistent, there is a large heterogeneity, which may reduce the reliability of meta-analysis results. Therefore, we conducted a meta-analysis of the above factors into subgroups to reduce the heterogeneity among the included studies. The results of subgroup analysis showed that the BBB score of the exosome group was significantly better than that of the negative control group, and the heterogeneity among the included studies was significantly reduced (Table 1). Therefore, our results are reliable.

**Table 1:** Results of subgroup analysis of BBB score (number of studies in brackets).

**Species**	**SCI models**	**Source**	**Transplant route**	**Transplant dose**	**WMD**	***I*** **^2^**
SD rats (27)	Contusion (23)	BMSCs-Exo (21)	Tail veins (19)	100 μg (8)	1 week: 1.47 [0.30, 2.65] 2 weeks: 2.26 [1.04, 3.49] 3 weeks: 2.48 [0.27, 4.70] 4 weeks: 3.72 [1.81, 5.63]	24.0% 20.5% 26.6% 25.4%
SD rats (27)	Contusion (23)	BMSCs-Exo (21)	Tail veins (19)	200 μg (11)	1 week: 1.76 [1.16, 2.36] 2 weeks: 2.98 [2.33, 3.63] 3 weeks: 3.74 [2.93, 4.56] 4 weeks: 4.36 [3.46, 5.26]	16.9% 18.3% 15.5% 13.9%
SD rats (27)	Contusion (23)	BMSCs-Exo (21)	Subcutaneous (1)			
SD rats (27)	Contusion (23)	BMSCs-Exo (21)	Not reported (1)			
SD rats (27)	Contusion (23)	NSCs-Exo (2)	Tail veins (2)	20 μg (1)		
SD rats (27)	Contusion (23)	NSCs-Exo (2)	Tail veins (2)	Not reported (1)		
SD rats (27)	Transection (3)	BMSCs-Exo (2)	Intranasal (1)			
SD rats (27)	Transection (3)	BMSCs-Exo (2)	Tail veins (1)			
SD rats (27)	Transection (3)	HpMSC-Exo (1)				
SD rats (27)	Not reported (1)					
C57BL/6 mice (3)	Not reported (3)					
Wistar rats (2)	Hemi-sectioned (1)					
Wistar rats (2)	Contusion (1)					

#### Apoptotic Protein and Antiapoptotic Protein

**Figure 5:**
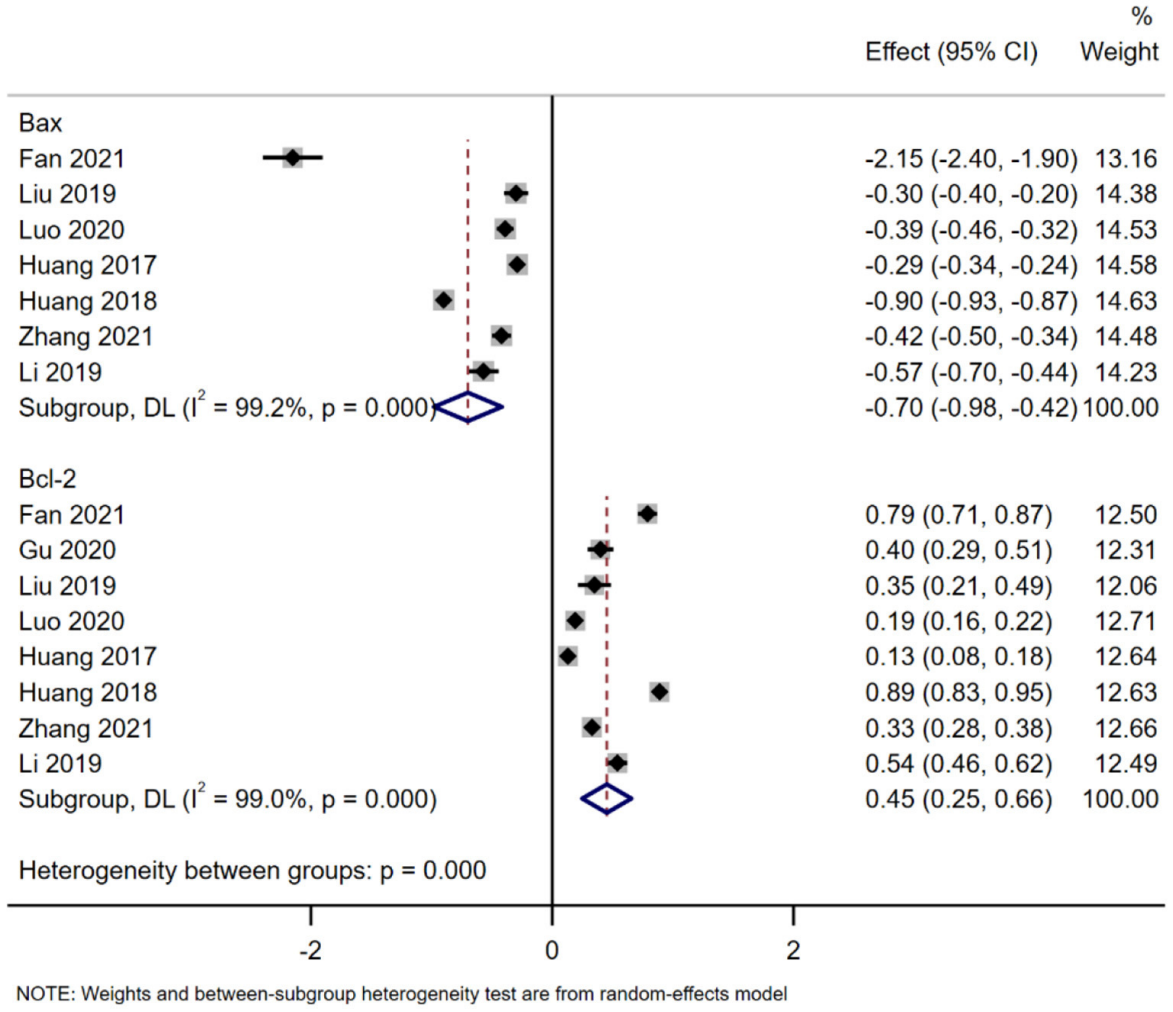
Meta-analysis results of BAX and Bcl-2.

A total of seven studies reported the expression level of BAX (31, 34, 35, 41, 46, 47, 53). The meta-analysis results of the random effects model showed that the expression level of BAX of the exosome group was significantly lower than that of the negative control group, and the difference was statistically significant [WDM = −0.70 (−0.98, −0.42), Figure 5]. A total of eight studies reported the expression level of Bcl-2. The meta-analysis results of the random effects model showed that the expression level of Bcl-2 of the exosome group was significantly better than that of the negative control group, and the difference was statistically significant [WDM = 0.45 (0.25, 0.66), Figure 5]. Subgroup analysis is impossible due to the limited number of included studies.

#### Pro-inflammatory Factors

**Figure 6:**
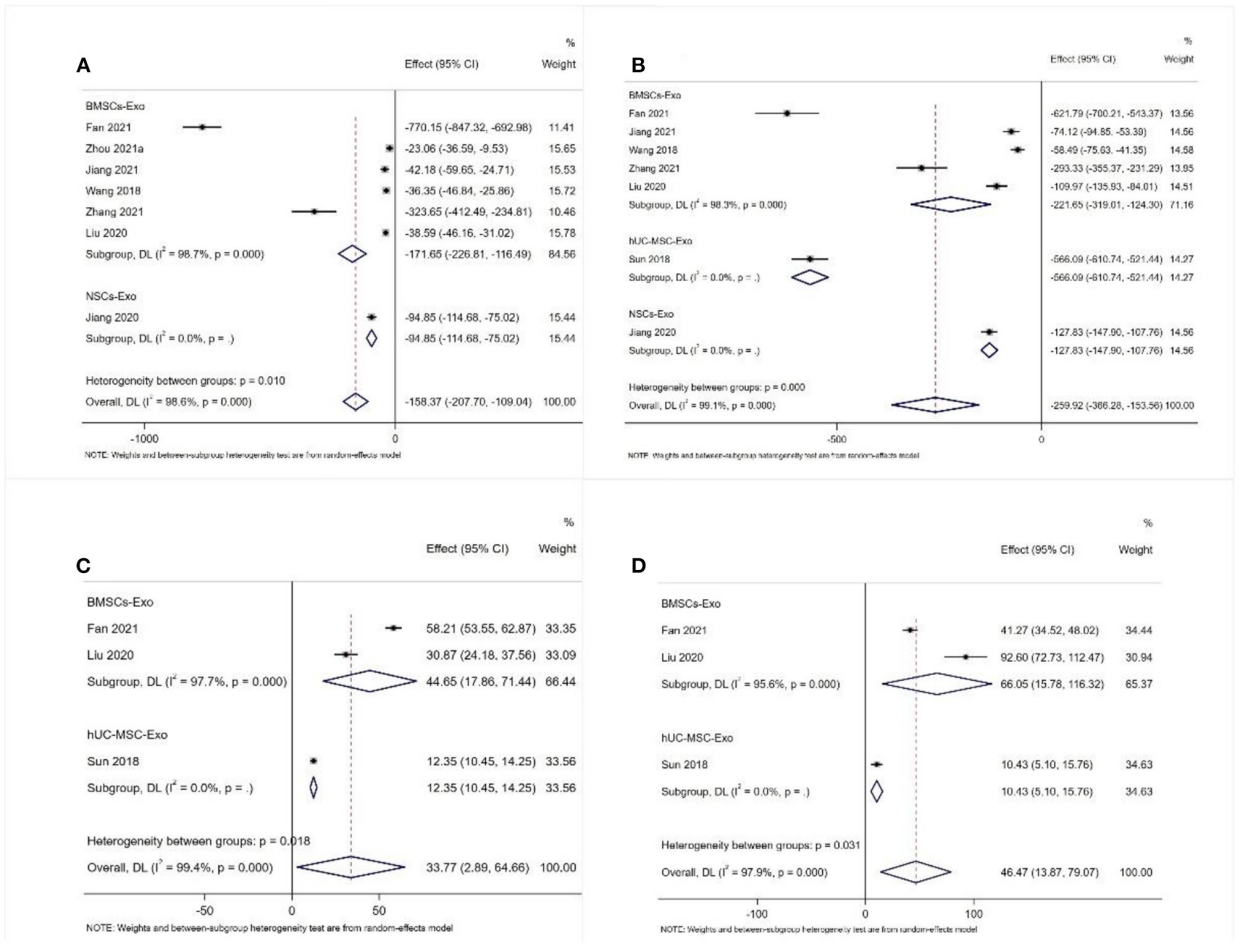
Meta-analysis results of pro-inflammatory factors and anti-inflammatory factors **(A)** IL-1β; **(B)** TNF-α; **(C)** IL-4; **(D)** IL-10.

A total of seven studies reported the expression level of IL-1β (31, 38, 39, 45, 49, 53, 56). Also, a total of seven studies reported the expression level of TNF-α (31, 38, 39, 45, 48, 49, 53). The meta-analysis results of the random effects model showed that the expression level of IL-1β and TNF-α of the exosome group was significantly lower than that of the negative control group, and the difference was statistically significant [IL-1β: WDM = −158.37 (−207.70, −109.04), Figure 6A; TNF-α: WDM = −259.92 (−336.28, −153.56), Figure 6B].

#### Anti-inflammatory Factor

A total of three studies reported the expression level of IL-4 and IL-10 (31, 45, 48). The meta-analysis results of the random effects model showed that the expression level of IL-4 and IL-10 of the exosome group was significantly better than that of the negative control group, and the difference was statistically significant [IL-4: WDM = 33.77 (2.89, 64.66), Figure 6C; IL-10: WDM = 46.47 (13.87, 79.07), Figure 6D].

## Discussion

### Overview of Evidence

The recovery of sensory and motor functions is crucial for patients with SCI. Our meta-analysis results indicated that exosomes derived from different stem cells can significantly improve the motor function of animals with SCI. The possible reason is that exosomes can improve the integrity of the blood-brain barrier (57) and inhibit neuronal apoptosis and necrosis (32). Based on the BBB score results at different time points, we found that the effect size of the BBB scores increased from the 1st to 4th weeks, indicating that the effect of exosome treatment gradually increased with time. The possible reason was that the exosome circulated in the body for a long time, and so the effect time was also long. Of course, the sustained repair role of endogenous stem cell activation on SCI also could not be ruled out (58). Although our study evaluates the therapeutic effect of exosomes as much as possible, the current study includes only 4-week follow-up data, and it is unknown whether the exosomes still perform well over a longer period of time. Therefore, future studies should further extend the follow-up time to observe whether or when exosomes can promote the complete recovery of motor function in SCI animals. In addition, while a direct comparison of exosomes of different stem cell sources is currently lacking, we could draw from the results of subgroup analysis that HpMSC-Exo and hUC-MSC-Exo are superior to BMSCs-Exo and NSCs-Exo in promoting motor function recovery in animals with SCI. However, fewer studies were included in subgroup analysis, which reduced the reliability of meta-analysis results. Therefore, more studies are needed to explore the efficacy of exosomes from different stem cell sources in repairing SCI to determine the best source of exosomes and promote the translation of animal experimental results to clinical practice. Meanwhile, a direct comparison of efficacy between exosomes of different stem cells is also an aspect of future studies. In addition, there may be great differences in the efficacy of different doses of exosome transplantation to promote the recovery of SCI. For example, Sun et al. compared the results of 200 and 20 μg of exosome transplantation doses and found that the motor function score of the 200 μg exosome group was significantly higher than that of the 20 μg exosome group, and there was no significant difference between the 20 μg exosome group and the PBS group (48). Therefore, the optimal transplantation dose of exosomes should also be of concern in the future.

The pathophysiological mechanism of SCI is very complicated, including apoptosis, inflammation, vascular injury, excitotoxicity, electrolyte disturbance, and mitochondrial dysfunction (35). Among them, apoptosis and inflammation are the two main events of secondary injury after SCI. The degree of apoptosis affects the functional recovery of SCI, which plays an important role in neuronal survival and axon regeneration (59). Apoptosis is mainly regulated by the upstream Bcl-2 family and the downstream caspase family, where anti- (Bcl-2) or pro-apoptotic (BAX) molecules are the most common apoptotic markers of programmed cell death, affecting the recovery of function after SCI (35, 60). Previous studies have shown that SCI promotes BAX expression and inhibits Bcl-2 expression (61, 62). In our study, the protein expression levels of apoptotic proteins (BAX) were significantly lower in the Exo group than in the negative group, while the anti-apoptotic protein (Bcl-2) level was observed to be significantly elevated. This suggests that exosomes have a protective effect on neuronal apoptosis induced by SCI (29).

In addition to the neuronal apoptosis, the blood-brain barrier of animals after SCI is destroyed. Neutrophils and M1 macrophages in the blood can rapidly infiltrate the damaged spinal cord tissue and secrete a large number of inflammatory factors, such as TNF-α, IL-1β, and IL-6 (63, 64). Increased levels of TNF- and IL-1 can be detected as early as 15 min after SCI, causing further SCI through its involvement in responses such as inflammation, demyelination, neuronal apoptosis, astrocyte toxicity, excitotoxicity, and oligodendrocyte death (2). In contrast, M2 macrophages suppress the inflammatory immune response by producing IL-4 and IL-10 cytokines (65, 66). The anti-inflammatory factor IL-10 can reduce the TNF-α produced by astrocytes and the antigen presentation of astrocytes and microglia, and significantly improve the functional recovery of animals after SCI (67, 68). Consistent with the results of animal studies, our meta-analysis showed that the secretion levels of anti-inflammatory factors were significantly higher in the exosome group than in the negative controls, and instead, the levels of pro-inflammatory factors were significantly lower than in the negative controls (31, 38, 39, 45, 48, 49, 53). We found that exosome transplantation can significantly reduce the apoptosis and inflammatory response of SCI animals, which is essential to promote the recovery of sensory and motor functions and prevent secondary SCI.

In conclusion, through the strict systematic review, we found that stem cell-derived exosomes have anti-inflammatory and anti-apoptotic effects and can significantly improve the motor function of animals with SCI. Therefore, the therapeutic potential of exosomes in SCI is enormous. Despite the success of exosome therapy for SCI in animal disease models, there are still some potential issues that need to be addressed before this approach can be translated into clinical applications due to the vast physiological differences between animals and humans. For example, future animal studies should further extend the follow-up time of animals and explore the therapeutic effect of exosomes over a longer period of time. At the same time, the therapeutic effect of different stem cell-derived exosomes should also be compared, and the types of exosomes with the most potential should be explored, as well as the best treatment strategies of exosomes, such as the best transplantation route, dose, and timing.

### Quality of Evidence

Based on the strict systematic review, our study found that the current animal experimental evidence quality of stem cell repair of SCI was not high, which reduced the reliability of the experimental results to a certain extent.

#### There Was Heterogeneity in the Included Studies

1. Inconsistencies in the definition of exosomes in different studies, such as the diameter of 20–130 nm extracted by Huang et al. (35) and Huang W et al. as 30–200 nm (21).
2. Exosome types (BMSCs, hUCMSCs, HpMSCs, and NSCs), sources (allogeneic animals or human tissues), transplantation route (intranasal, intravenous, subcutaneous transplantation), transplantation timing (immediately to 24 h after surgery), and transplantation dose (20–200 μg) are inconsistent.
3. The baseline characteristics of animal species (SD rats, Wistar rats, C57/BL6 mice), age (6–12 weeks), body weight (17–300 g), and models (contusion, hemisection, and transection) are inconsistent.
4. There was also inconsistency in the outcome measures. Especially for BBB scores, although its 21 entries clearly illustrate how to score, differences in familiarity and understanding of BBB score criteria among different experimental researchers can lead to differences in scores obtained on the same degree of SCI between different studies, ultimately affecting the reliability of the results (69).

Although our subgroup analysis has reduced the heterogeneity among included studies to some extent, the test efficacy of the subgroup analysis was limited by the small number of studies. In conclusion, the limitation of included interstudy heterogeneity somewhat reduces the reliability of the results of our meta-analysis.

#### Insufficient Intrinsic Authenticity of Included Studies

##### Selection Bias

Although the baseline characteristics of animals in the same study, such as age, gender, and weight, are balanced, the baseline characteristics of animals in different studies are quite different. In addition, the included studies have not reported the details of the random allocation of animals to the experimental group and the control group. Therefore, we cannot determine whether the random grouping method is correct. At the same time, there is no research report on whether allocation concealment was implemented; therefore, there is a certain selective bias in the included studies. Future research needs to further standardize the grouping of animals and report the implementation details of experiments in accordance with the ARRIVE guidelines so as to improve the quality of the report of animal experiments (70).

##### Implementation Bias

Although 78.13% (25/32) of the studies reported randomized placement of animals during the breeding process, none of the studies could determine whether to blind animal breeders and researchers. Although there is no need to blind animals, it is necessary to blind the experimenters to avoid the introduction of implementation bias due to subjective factors in the implementation stage of intervention measures.

##### Measurement Bias

Only 59.38% (19/32) of the studies blinded the result evaluators, and 6% (2/32) of the studies randomly selected animals during the evaluation of the results, leading to a certain measurement bias in the included studies. Therefore, future studies should focus more on the application of randomization and blinding in outcome evaluation to improve the repeatability and reliability of animal experiments (71).

##### Report Bias

Protocols were not available for all studies, and it cannot be determined whether all of their experimental results will be reported without bias. Selective reporting of results can lead to publication bias, which affects the reliability of experimental results (72). Although it is difficult to register and report protocols of animal experiments, we still encourage animal experiment researchers to register experiment protocols forward-looking to avoid publication bias caused by selectively reporting animal experiment research results (72).

##### Publication Bias

Experiments with positive results are generally more likely to be published than those with negative or invalid results (73). Publication bias may be more serious in animal experiments (74). Systematic reviews that do not include unpublished studies are likely to overestimate the effects of interventions. The asymmetric funnel plot suggests that the current study may be subject to publication bias to some extent. In conclusion, in the field of experimental research, it is necessary to take measures to promote data sharing and develop policies to encourage and require journals to publish studies with negative or neutral results in order to avoid the “drawer document” effect and reduce the impact of publication bias on their results (75).

##### Quality of Evidence

The overall quality of evidence is a key factor affecting decision-making. Due to the large inherent bias risk and publication bias in the current animal experiments, as well as the inaccuracy of the results (mainly the wide confidence interval), the credibility of our meta-analysis results is greatly reduced. Therefore, future animal experiments should follow the ARRIVE guidelines and SYRCLE's risk of bias tool, improve the evidence quality of animal experiments as much as possible, and promote the clinical translation of exosome therapy for SCI as soon as possible.

#### Insufficient External Authenticity of Included Studies

External authenticity refers to the extent to which clinical trial results can be reproduced repeatedly in the target population and daily population (76). For animal studies, external authenticity mainly relates to the reproducibility of animal experiments and the feasibility of transforming animal experiment results into clinical practice.

1. Through a comprehensive evaluation of the included studies, we found that the internal authenticity of current animal experiments is insufficient, and the quality of animal experiments reports needs to be improved, which reduces the reliability and repeatability of animal experiments to some extent.
2. In animal studies, the efficacy of exosomes can only be explored by BBB score, apoptosis protein, inflammatory factors, and other relatively objective outcome indicators. However, the subtle changes in sensory function, nerve root movement, and pain that are clinically concerned cannot be fully studied by animal studies.
3. There are various sources of exosomes in animal experiments, but in clinical research, due to safety and ethical restrictions, exosomes can only come from human tissues.
4. In animal studies, there are various models of SCI (including contusion, transection, and hemisection), and the injury sites are all thoracic spine or lumbar spine. In clinical research, cervical spine injury is more common (77). Meanwhile, experimental animals, in contrast to human patients, are injured under controlled conditions with a simple wound to a targeted spinal region and are more uniform and less complex. Generally, people suffer from multiple injuries, such as fractures of vertebrae, or even accompanying traumatic injury of the brain (78).
5. In clinical studies, patients with SCI may have debilitating states such as aging and diabetes that may decrease the ability of exosomes to promote neural regeneration and angiogenesis, while it is difficult to simultaneously simulate the debilitating state in animal studies (79).
6. The safety of a drug or treatment is the prerequisite for its clinical application, followed by its effectiveness (80). However, except for Huang et al. (20), few studies evaluated the side effects of exosomes, such as hepatotoxicity. Therefore, future research should also pay attention to the side effects of exosome transplantation.

In short, due to the abovementioned external authenticity restrictions, the feasibility of the current animal experimental results to be transformed into clinical practice is reduced.

### Strengths and Limitations of the Present Study

Key strengths of this systematic review: (1) We comprehensively evaluated and analyzed the role of exosomes in SCI and the limitations of current animal experiments while pointing out the problems and directions for improvement. This is the first time in the current field. (2) We comprehensively evaluated the therapeutic effect of exosomes in SCI from the macro (BBB score) and micro (apoptotic proteins, inflammatory factors) perspectives, and conducted subgroup analysis of the research results to increase the reliability of the results. (3) Based on the internationally recognized SYRCLE bias risk assessment tool, the internal bias risk of animal studies was strictly evaluated, and the problems in the design and implementation of animal studies in this field were pointed out. At the same time, suggestions on how to improve the quality of animal experiments were given. This is essential to improve the quality of future animal experiments and explore the therapeutic effect of exosomes more accurately. (4) Based on the GRADE evidence grading system, the evidence quality of each outcome indicator was evaluated, and the feasibility of transforming animal studies to clinical trials was evaluated more scientifically.

Limitations of this systematic review: (1) We determine whether the study was included in our comprehensive analysis based on the definition of exosomes in animal studies. It may be excluded that some exosomes are not well-defined, but the intervention is the study of exosomes. (2) For the outcome indicators such as apoptotic proteins and inflammatory factors, most studies only reported the results at the end of the follow-up, and there were significant differences. Therefore, our data at different time points were combined, resulting in greater heterogeneity and reducing the reliability of meta-analysis results. (3) We could not accurately identify the source of heterogeneity; therefore, we adopted a random-effects model for meta-analysis, making our conclusions more conservative. (4) Only Chinese and English databases were retrieved, which may lead to a certain language bias. (5) Gray literature and conference abstracts were not searched, potentially leading to the generation of publication bias.

## Conclusion

Through the comprehensive analysis of the 32 studies included, we found that exosomes from different stem cells could significantly improve the motor function of animals with SCI, increase the levels of anti-apoptotic proteins and anti-inflammatory factors, and reduce the levels of apoptotic proteins and pro-inflammatory factors. We also found that the repair effect of exosomes derived from human tissues is better than that of exosomes derived from animal tissues, and the longer the treatment time of exosomes, the more significant the improvement effect on SCI.

However, through a comprehensive analysis of the quality of evidence, internal authenticity and external authenticity of the included studies, we believed that current animal studies still have certain problems in randomization, allocation concealment, blinding, and result from measurement and reporting. Therefore, future research needs to further standardize the implementation and reporting of animal studies to improve the quality of evidence in preclinical research and promote better the translation of preclinical research results into clinical practice.

## Data Availability Statement

The original contributions presented in the study are included in the article/Supplementary Material, further inquiries can be directed to the corresponding author.

## Author Contributions

XK undertook the design, guidance, and modification of the project and manuscript. CZ completed the implementation of the project and writing of the manuscript. RD, GZ, XH, HC, BC, LW, and XK completed the collection and collation of the data. All authors contributed to the article and approved the submitted version.

## Conflict of Interest

The authors declare that the research was conducted in the absence of any commercial or financial relationships that could be construed as a potential conflict of interest.

## Publisher's Note

All claims expressed in this article are solely those of the authors and do not necessarily represent those of their affiliated organizations, or those of the publisher, the editors and the reviewers. Any product that may be evaluated in this article, or claim that may be made by its manufacturer, is not guaranteed or endorsed by the publisher.

## References

[1] Lattard A Poulen G Bartolami S Gerber YN Perrin FE. Negative impact of sigma-1 receptor agonist treatment on tissue integrity and motor function following spinal cord injury. Front Pharmacol. (2021) 12:614949. 10.3389/fphar.2021.614949 33643047 PMC7902910

[2] Ahuja CS Wilson JR Nori S Kotter MRN Druschel C Curt A . Traumatic spinal cord injury. Nat Rev Dis Primers. (2017) 3:17018. 10.1038/nrdp.2017.18 28447605

[3] Ramer LM Ramer MS Bradbury EJ. Restoring function after spinal cord injury: towards clinical translation of experimental strategies. Lancet Neurol. (2014) 13:1241–56. 10.1016/S1474-4422(14)70144-9 25453463

[4] Tran AP Warren PM Silver J. The biology of regeneration failure and success after spinal cord injury. Physiol Rev. (2018) 98:881–917. 10.1152/physrev.00017.2017 29513146 PMC5966716

[5] Jeong JO Han JW Kim JM Cho HJ Park C Lee N . Malignant tumor formation after transplantation of short-term cultured bone marrow mesenchymal stem cells in experimental myocardial infarction and diabetic neuropathy. Circ Res. (2011) 108:1340–7. 10.1161/CIRCRESAHA.110.239848 21493893 PMC3109741

[6] Koprivec S Novak M Bernik S Voga M Mohorič L Majdič D. Treatment of cranial cruciate ligament injuries in dogs using a combination of tibial tuberosity advancement procedure and autologous mesenchymal stem cells/multipotent mesenchymal stromal cells - a pilot study. Acta Vet Hung. (2021) 68:405–12. 10.1556/004.2020.00063 33656452

[7] Liau LL Looi QH Chia WC Subramaniam T Ng MH Law JX. Treatment of spinal cord injury with mesenchymal stem cells. Cell Biosci. (2020) 10:112. 10.1186/s13578-020-00475-3 32983406 PMC7510077

[8] Phinney DG Prockop DJ. Concise review: mesenchymal stem/multipotent stromal cells: the state of transdifferentiation and modes of tissue repair–current views. Stem Cells. (2007) 25:2896–902. 10.1634/stemcells.2007-0637 17901396

[9] Keshtkar S Azarpira N Ghahremani MH. Mesenchymal stem cell-derived extracellular vesicles: novel frontiers in regenerative medicine. Stem Cell Res Ther. (2018) 9:63. 10.1186/s13287-018-0791-7 29523213 PMC5845209

[10] Aghajani Nargesi A Lerman LO Eirin A. Mesenchymal stem cell-derived extracellular vesicles for kidney repair: current status and looming challenges. Stem Cell Res Ther. (2017) 8:273. 10.1186/s13287-017-0727-7 29202871 PMC5713024

[11] Cao Y Xu Y Chen C Xie H Lu H Hu J. Local delivery of USC-derived exosomes harboring ANGPTL3 enhances spinal cord functional recovery after injury by promoting angiogenesis. Stem Cell Res Ther. (2021) 12:20. 10.1186/s13287-020-02078-8 33413639 PMC7791988

[12] Lener T Gimona M Aigner L Börger V Buzas E Camussi G . Applying extracellular vesicles based therapeutics in clinical trials - an ISEV position paper. J Extracell Vesicles. (2015) 4:30087. 10.3402/jev.v4.30087 26725829 PMC4698466

[13] Zheng G Huang R Qiu G Ge M Wang J Shu Q . Mesenchymal stromal cell-derived extracellular vesicles: regenerative and immunomodulatory effects and potential applications in sepsis. Cell Tissue Res. (2018) 374:1–15. 10.1007/s00441-018-2871-5 29955951

[14] Huang CC Kang M Lu Y Shirazi S Diaz JI Cooper LF . Functionally engineered extracellular vesicles improve bone regeneration. Acta Biomater. (2020) 109:182–94. 10.1016/j.actbio.2020.04.017 32305445 PMC8040700

[15] Bari E Perteghella S Di Silvestre D Sorlini M Catenacci L Sorrenti M . Pilot production of mesenchymal stem/stromal freeze-dried secretome for cell-free regenerative nanomedicine: a validated GMP-compliant process. Cells. (2018) 7:110190. 10.3390/cells7110190 30380806 PMC6262564

[16] He C Zheng S Luo Y Wang B. Exosome theranostics: biology and translational medicine. Theranostics. (2018) 8:237–55. 10.7150/thno.21945 29290805 PMC5743472

[17] Ouyang X Han X Chen Z Fang J Huang X Wei H. MSC-derived exosomes ameliorate erectile dysfunction by alleviation of corpus cavernosum smooth muscle apoptosis in a rat model of cavernous nerve injury. Stem Cell Res Ther. (2018) 9:246. 10.1186/s13287-018-1003-1 30257719 PMC6158845

[18] Doeppner TR Herz J Görgens A Schlechter J Ludwig AK Radtke S . Extracellular vesicles improve post-stroke neuroregeneration and prevent postischemic immunosuppression. Stem Cells Transl Med. (2015) 4:1131–43. 10.5966/sctm.2015-0078 26339036 PMC4572905

[19] Li L Zhang Y Mu J Chen J Zhang C Cao H . Transplantation of human mesenchymal stem-cell-derived exosomes immobilized in an adhesive hydrogel for effective treatment of spinal cord injury. Nano Lett. (2020) 20:4298–305. 10.1021/acs.nanolett.0c00929 32379461

[20] Huang W Qu M Li L Liu T Lin M Yu X . in MSC-derived exosomes silences CTGF gene for locomotor recovery in spinal cord injury rats. Stem Cell Res Ther. (2021) 12:334. 10.1186/s13287-021-02401-x 34112262 PMC8193895

[21] Huang W Lin M Yang C Wang F Zhang M Gao J . Rat Bone mesenchymal stem cell-derived exosomes loaded with miR-494 promoting neurofilament regeneration and behavioral function recovery after spinal cord injury. Oxid Med Cell Longev. (2021) 2021:1634917. 10.1155/2021/1634917 34635862 PMC8501401

[22] Chen CC Liu L Ma F Wong CW Guo XE Chacko JV . Elucidation of exosome migration across the blood-brain barrier model *in vitro*. Cell Mol Bioeng. (2016) 9:509–29. 10.1007/s12195-016-0458-3 28392840 PMC5382965

[23] Willis GR Fernandez-Gonzalez A Anastas J Vitali SH Liu X Ericsson M . Mesenchymal stromal cell exosomes ameliorate experimental bronchopulmonary dysplasia and restore lung function through macrophage immunomodulation. Am J Respir Crit Care Med. (2018) 197:104–16. 10.1164/rccm.201705-0925OC 28853608 PMC5765387

[24] Hessvik NP Llorente A. Current knowledge on exosome biogenesis and release. Cell Mol Life Sci. (2018) 75:193–208. 10.1007/s00018-017-2595-9 28733901 PMC5756260

[25] Basso DM Beattie MS Bresnahan JC Anderson DK Faden AI Gruner JA . MASCIS evaluation of open field locomotor scores: effects of experience and teamwork on reliability. Multicenter animal spinal cord injury study. J Neurotr. (1996) 13:343–59. 10.1089/neu.1996.13.343 8863191

[26] Hooijmans CR Rovers MM de Vries RB Leenaars M Ritskes-Hoitinga M Langendam MW. SYRCLE's risk of bias tool for animal studies. BMC Med Res Methodol. (2014) 14:43. 10.1186/1471-2288-14-43 24667063 PMC4230647

[27] Guyatt GH Oxman AD Vist GE Kunz R Falck-Ytter Y Alonso-Coello P . an emerging consensus on rating quality of evidence and strength of recommendations. BMJ. (2008) 336:924–6. 10.1136/bmj.39489.470347.AD 18436948 PMC2335261

[28] Chang Q Hao Y Wang Y Zhou Y Zhuo H Zhao G. Bone marrow mesenchymal stem cell-derived exosomal microRNA-125a promotes M2 macrophage polarization in spinal cord injury by downregulating IRF5. Brain Res Bull. (2021) 170:199–210. 10.1016/j.brainresbull.2021.02.015 33609602

[29] Chen J Zhang C Li S Li Z Lai X Xia Q. Exosomes derived from nerve stem cells loaded with FTY720 promote the recovery after spinal cord injury in rats by PTEN/AKT signal pathway. J Immunol Res. (2021) 2021:8100298. 10.1155/2021/8100298 34337080 PMC8294984

[30] Chen Y Tian Z He L Liu C Wang N Rong L . Exosomes derived from miR-26a-modified MSCs promote axonal regeneration via the PTEN/AKT/mTOR pathway following spinal cord injury. Stem Cell Res Ther. (2021) 12:224. 10.1186/s13287-021-02282-0 33820561 PMC8022427

[31] Fan L Dong J He X Zhang C Zhang T. Bone marrow mesenchymal stem cells-derived exosomes reduce apoptosis and inflammatory response during spinal cord injury by inhibiting the TLR4/MyD88/NF-κB signaling pathway. Hum Exp Toxicol. (2021) 40:1612–23. 10.1177/09603271211003311 33779331

[32] Gu J Jin ZS Wang CM Yan XF Mao YQ Chen S. Bone marrow mesenchymal stem cell-derived exosomes improves spinal cord function after injury in rats by activating autophagy. Drug Des Devel Ther. (2020) 14:1621–31. 10.2147/DDDT.S237502 32425507 PMC7196809

[33] Guo S Perets N Betzer O Ben-Shaul S Sheinin A Michaelevski I . Intranasal delivery of mesenchymal stem cell derived exosomes loaded with phosphatase and tensin homolog siRNA repairs complete spinal cord injury. ACS Nano. (2019) 13:10015–28. 10.1021/acsnano.9b01892 31454225

[34] Huang JH Xu Y Yin XM Lin FY. Exosomes derived from miR-126-modified MSCs promote angiogenesis and neurogenesis and attenuate apoptosis after spinal cord injury in rats. Neuroscience. (2020) 424:133–45. 10.1016/j.neuroscience.2019.10.043 31704348

[35] Huang JH Yin XM Xu Y Xu CC Lin X Ye FB . Systemic administration of exosomes released from mesenchymal stromal cells attenuates apoptosis, inflammation, and promotes angiogenesis after spinal cord injury in rats. J Neurotrauma. (2017) 34:3388–96. 10.1089/neu.2017.5063 28665182

[36] Jia Y Lu T Chen Q Pu X Ji L Yang J . Exosomes secreted from sonic hedgehog-modified bone mesenchymal stem cells facilitate the repair of rat spinal cord injuries. Acta Neurochir. (2021) 163:2297–306. 10.1007/s00701-021-04829-9 33821317 PMC8270837

[37] Jia Y Yang J Lu T Pu X Chen Q Ji L . Repair of spinal cord injury in rats via exosomes from bone mesenchymal stem cells requires sonic hedgehog. Regenerative therapy. (2021) 18:309–15. 10.1016/j.reth.2021.08.007 34522723 PMC8416644

[38] Jiang D Gong F Ge X Lv C Huang C Feng S . Neuron-derived exosomes-transmitted miR-124-3p protect traumatically injured spinal cord by suppressing the activation of neurotoxic microglia and astrocytes. J Nanobiotechnology. (2020) 18:105. 10.1186/s12951-020-00665-8 32711535 PMC7382861

[39] Jiang Z Zhang J. Mesenchymal stem cell-derived exosomes containing miR-145-5p reduce inflammation in spinal cord injury by regulating the TLR4/NF-κB signaling pathway. Cell Cycle. (2021) 20:993–1009. 10.1080/15384101.2021.1919825 33945431 PMC8172161

[40] Kang J Zhang C Zhi Z Wang Y Liu J Wu F . Stem-like cells of various origins showed therapeutic effect to improve the recovery of spinal cord injury. Artificial Cells Nanomed Biotechnol. (2020) 48:627–38. 10.1080/21691401.2020.1725031 32054316

[41] Li C Jiao G Wu W Wang H Ren S Zhang L . Exosomes from bone marrow mesenchymal stem cells inhibit neuronal apoptosis and promote motor function recovery *via* the Wnt/β-catenin signaling pathway. Cell Transplant. (2019) 28:1373–83. 10.1177/0963689719870999 31423807 PMC6802144

[42] Li C Li X Zhao B Wang C. Exosomes derived from miR-544-modified mesenchymal stem cells promote recovery after spinal cord injury. Arch Physiol Biochem. (2020) 126:369–75. 10.1080/13813455.2019.1691601 32141339

[43] Li D Zhang P Yao X Li H Shen H Li X . Exosomes derived from miR-133b-modified mesenchymal stem cells promote recovery after spinal cord injury. Front Neurosci. (2018) 12:845. 10.3389/fnins.2018.00845 30524227 PMC6262643

[44] Liu J Lin M Qiao F Zhang C. Exosomes derived from lncRNA TCTN2-modified mesenchymal stem cells improve spinal cord injury by miR-329-3p/IGF1R axis. J Mol Neurosci. (2021) 1–14. 10.1007/s12031-021-01914-7 34623606

[45] Liu W Rong Y Wang J Zhou Z Ge X Ji C . Exosome-shuttled miR-216a-5p from hypoxic preconditioned mesenchymal stem cells repair traumatic spinal cord injury by shifting microglial M1/M2 polarization. J Neuroinflammation. (2020) 17:47. 10.1186/s12974-020-1726-7 32019561 PMC7001326

[46] Liu W Wang Y Gong F Rong Y Luo Y Tang P . Exosomes derived from bone mesenchymal stem cells repair traumatic spinal cord injury by suppressing the activation of A1 neurotoxic reactive astrocytes. J Neurotrauma. (2019) 36:469–84. 10.1089/neu.2018.5835 29848167

[47] Luo Y Xu T Liu W Rong Y Wang J Fan J . Exosomes derived from GIT1-overexpressing bone marrow mesenchymal stem cells promote traumatic spinal cord injury recovery in a rat model. Int J Neurosci. (2021) 131:170–82. 10.1080/00207454.2020.1734598 32223487

[48] Sun G Li G Li D Huang W Zhang R Zhang H . hucMSC derived exosomes promote functional recovery in spinal cord injury mice *via* attenuating inflammation. Mater Sci Eng C Mater Biol Appl. (2018) 89:194–204. 10.1016/j.msec.2018.04.006 29752089

[49] Wang L Pei S Han L Guo B Li Y Duan R . Mesenchymal stem cell-derived exosomes reduce A1 astrocytes *via* downregulation of phosphorylated NFκB P65 subunit in spinal cord injury. Cell Physiol Biochem. (2018) 50:1535–59. 10.1159/000494652 30376671

[50] Wang Y Lai X Wu D Liu B Wang N Rong L. Umbilical mesenchymal stem cell-derived exosomes facilitate spinal cord functional recovery through the miR-199a-3p/145-5p-mediated NGF/TrkA signaling pathway in rats. Stem Cell Res Ther. (2021) 12:117. 10.1186/s13287-021-02148-5 33579361 PMC7879635

[51] Xin W Qiang S Jianing D Jiaming L Fangqi L Bin C . Human bone marrow mesenchymal stem cell-derived exosomes attenuate blood-spinal cord barrier disruption *via* the TIMP2/MMP pathway after acute spinal cord injury. Mol Neurobiol. (2021) 58:6490–504. 10.1007/s12035-021-02565-w 34554399

[52] Yu T Zhao C Hou S Zhou W Wang B Chen Y. Exosomes secreted from miRNA-29b-modified mesenchymal stem cells repaired spinal cord injury in rats. Brazil J Med Biol Res. (2019) 52:e8735. 10.1590/1414-431x20198735 31826179 PMC6903804

[53] Zhang M Wang L Huang S He X. Exosomes with high level of miR-181c from bone marrow-derived mesenchymal stem cells inhibit inflammation and apoptosis to alleviate spinal cord injury. J Mol Histol. (2021) 52:301–11. 10.1007/s10735-020-09950-0 33548000

[54] Zhao C Zhou X Qiu J Xin D Li T Chu X . Exosomes derived from bone marrow mesenchymal stem cells inhibit complement activation in rats with spinal cord injury. Drug Des Devel Ther. (2019) 13:3693–704. 10.2147/DDDT.S209636 31695336 PMC6817353

[55] Zhou W Silva M Feng C Zhao S Liu L Li S . Exosomes derived from human placental mesenchymal stem cells enhanced the recovery of spinal cord injury by activating endogenous neurogenesis. Stem Cell Res Ther. (2021) 12:174. 10.1186/s13287-021-02248-2 33712072 PMC7953814

[56] Zhou Y Wen LL Li YF Wu KM Duan RR Yao YB . Exosomes derived from bone marrow mesenchymal stem cells protect the injured spinal cord by inhibiting pericyte pyroptosis. Neural Regen Res. (2022) 17:194–202. 10.4103/1673-5374.314323 34100456 PMC8451579

[57] Lu Y Zhou Y Zhang R Wen L Wu K Li Y . Bone mesenchymal stem cell-derived extracellular vesicles promote recovery following spinal cord injury *via* improvement of the integrity of the blood-spinal cord barrier. Front Neurosci. (2019) 13:209. 10.3389/fnins.2019.00209 30914918 PMC6423165

[58] Qin Y Zhang W Yang P. Current states of endogenous stem cells in adult spinal cord. J Neurosci Res. (2015) 93:391–8. 10.1002/jnr.23480 25228050

[59] Tang P Hou H Zhang L Lan X Mao Z Liu D . Autophagy reduces neuronal damage and promotes locomotor recovery via inhibition of apoptosis after spinal cord injury in rats. Mol Neurobiol. (2014) 49:276–87. 10.1007/s12035-013-8518-3 23954967

[60] Zhang J Cui Z Feng G Bao G Xu G Sun Y . RBM5 and p53 expression after rat spinal cord injury: implications for neuronal apoptosis. Int J Biochem Cell Biol. (2015) 60:43–52. 10.1016/j.biocel.2014.12.020 25578565

[61] Lin CL Wang JY Huang YT Kuo YH Surendran K Wang FS. Wnt/beta-catenin signaling modulates survival of high glucose-stressed mesangial cells. J Am Soc Nephrol. (2006) 17:2812–20. 10.1681/ASN.2005121355 16943306

[62] Adams JM Cory S. The Bcl-2 protein family: arbiters of cell survival. Science. (1998) 281:1322–6. 10.1126/science.281.5381.1322 9735050

[63] Brown GC. Mechanisms of inflammatory neurodegeneration: iNOS and NADPH oxidase. Biochem Soc Trans. (2007) 35:1119–21. 10.1042/BST0351119 17956292

[64] Bellora F Castriconi R Dondero A Reggiardo G Moretta L Mantovani A . The interaction of human natural killer cells with either unpolarized or polarized macrophages results in different functional outcomes. Proc Natl Acad Sci USA. (2010) 107:21659–64. 10.1073/pnas.1007654108 21118979 PMC3003022

[65] Kigerl KA Gensel JC Ankeny DP Alexander JK Donnelly DJ Popovich PG. Identification of two distinct macrophage subsets with divergent effects causing either neurotoxicity or regeneration in the injured mouse spinal cord. J Neurosci. (2009) 29:13435–44. 10.1523/JNEUROSCI.3257-09.2009 19864556 PMC2788152

[66] Mantovani A Sica A Sozzani S Allavena P Vecchi A Locati M. The chemokine system in diverse forms of macrophage activation and polarization. Trends Immunol. (2004) 25:677–86. 10.1016/j.it.2004.09.015 15530839

[67] Bethea JR Nagashima H Acosta MC Briceno C Gomez F Marcillo AE . Systemically administered interleukin-10 reduces tumor necrosis factor-alpha production and significantly improves functional recovery following traumatic spinal cord injury in rats. J Neurotrauma. (1999) 16:851–63. 10.1089/neu.1999.16.851 10547095

[68] Brewer KL Bethea JR Yezierski RP. Neuroprotective effects of interleukin-10 following excitotoxic spinal cord injury. Exp Neurol. (1999) 159:484–93. 10.1006/exnr.1999.7173 10506519

[69] Tator CH. Review of treatment trials in human spinal cord injury: issues, difficulties, and recommendations. Neurosurgery. (2006) 59:957–82. 10.1227/01.NEU.0000245591.16087.89 17143232

[70] Percie du Sert N Hurst V Ahluwalia A Alam S Avey MT Baker M . The ARRIVE guidelines 2.0: updated guidelines for reporting animal research. J Cerebr Blood Flow Metabol. (2020) 40:1769–77. 10.1177/0271678X20943823 32663096 PMC7430098

[71] Dell RB Holleran S Ramakrishnan R. Sample size determination. ILAR J. (2002) 43:207–13. 10.1093/ilar.43.4.207 12391396 PMC3275906

[72] Korevaar DA Hooft L ter Riet G. Systematic reviews and meta-analyses of preclinical studies: publication bias in laboratory animal experiments. Lab Anim. (2011) 45:225–30. 10.1258/la.2011.010121 21737463

[73] Scherer RW Meerpohl JJ Pfeifer N Schmucker C Schwarzer G von Elm E. Full publication of results initially presented in abstracts. Cochr Database Systemat Rev. (2018) 11:Mr000005. 10.1002/14651858.MR000005.pub4 30480762 PMC7073270

[74] Ioannidis JP. Extrapolating from animals to humans. Sci Transl Med. (2012) 4:151ps15. 10.1126/scitranslmed.3004631 22972841

[75] Begley CG Ellis LM. Drug development: raise standards for preclinical cancer research. Nature. (2012) 483:531–3. 10.1038/483531a 22460880

[76] Kennedy-Martin T Curtis S Faries D Robinson S Johnston J. A literature review on the representativeness of randomized controlled trial samples and implications for the external validity of trial results. Trials. (2015) 16:1–14. 10.1186/s13063-015-1023-4 26530985 PMC4632358

[77] Dvorak MF Noonan VK Fallah N Fisher CG Rivers CS Ahn H . Minimizing errors in acute traumatic spinal cord injury trials by acknowledging the heterogeneity of spinal cord anatomy and injury severity: an observational Canadian cohort analysis. J Neurotrauma. (2014) 31:1540–7. 10.1089/neu.2013.3278 24811484 PMC4161054

[78] Sharif-Alhoseini M Khormali M Rezaei M Safdarian M Hajighadery A Khalatbari MM . Animal models of spinal cord injury: a systematic review. Spinal Cord. (2017) 55:714–21. 10.1038/sc.2016.187 28117332

[79] El-Ftesi S Chang EI Longaker MT Gurtner GC. Aging and diabetes impair the neovascular potential of adipose-derived stromal cells. Plast Reconstr Surg. (2009) 123:475–85. 10.1097/PRS.0b013e3181954d08 19182604 PMC2878769

[80] Ozdemir V Shear NH Kalow W. What will be the role of pharmacogenetics in evaluating drug safety and minimising adverse effects? Drug safety. (2001) 24:75–85. 10.2165/00002018-200124020-00001 11235820

