# Supplementary material for: Therapeutic Effect of Exosomes Derived From Stem Cells in Spinal Cord Injury: A Systematic Review Based on Animal Studies
Source: Front Neurol. 2022 Mar 10;13:847444. doi: 10.3389/fneur.2022.847444 (PMC8959939; doi:10.3389/fneur.2022.847444)
Supplement: Supplementary file 1 [file Data_Sheet_1.docx]

**Therapeutic Effect of Exosomes Derived from Stem Cells in Spinal Cord Injury: A Systematic Review Based on Animal Studies**

Cangyu Zhang^1,2^, Rongrong Deng, Guangzhi Zhang^1,2^, Xuegang He^1,2^, Haiwei Chen^1,2^, Bao Chen^1,2^, Lin Wan^1,2^, Xuewen Kang^1,2*^

1. Department of Orthopaedics, the Second Hospital of Lanzhou University, Lanzhou 730030, China

2. Key Laboratory of Osteoarthritis of Gansu Province, Lanzhou 730030, China

3. Department of Nephrology, the Second Hospital of Lanzhou University, Lanzhou 730030, China

*Corresponding author:

E-mail: ery_kangxw@lzu.edu.cn (Xuewen Kang)

**Table1: Detailed retrieval process for each database**

| **1.WOS**  (TS=(exosome OR exosomes OR secretome OR allochthon OR exosomal)) AND (TS=( spinal cord compression OR spinal cord contusions OR spinal cord injury OR spinal cord injuries OR spinal injuries OR spinal cord trauma OR spinal cord transection OR spinal cord laceration OR post traumatic myelopathy)) 302  2.PubMed  #1: "Exosomes"[MeSH Terms] 9660  #2: "exosome"[Title/Abstract] OR "exosomes"[Title/Abstract] OR "secretome"[Title/Abstract] OR "exosomal"[Title/Abstract] 23989  #3: #1 OR #2  #4: "Spinal Cord Injuries"[MeSH Terms] 51558  #5: "spinal cord compression"[Title/Abstract] OR "spinal cord contusions"[Title/Abstract] OR "spinal cord injury"[Title/Abstract] OR "spinal cord injuries"[Title/Abstract] OR "spinal injuries"[Title/Abstract] OR "spinal cord trauma"[Title/Abstract] OR "spinal cord transection"[Title/Abstract] OR "spinal cord laceration"[Title/Abstract] OR "post traumatic myelopathy"[Title/Abstract] 51048  #6: #4 OR #5 69210  #7: #3 AND #6 138  3.Ovid-Embase  #1: (exosome or exosomes or secretome or allochthon or exosomal).mp. [mp=title, abstract, heading word, drug trade name, original title, device manufacturer, drug manufacturer, device trade name, keyword heading word, floating subheading word, candidate term word] 47046  #2: exosome/ 38002  #3: 1 or 2 47046  #4: (spinal cord compression or spinal cord contusions or spinal cord injury or spinal cord injuries or spinal injuries or spinal cord trauma or spinal cord transection or spinal cord laceration or post traumatic myelopathy).mp. [mp=title, abstract, heading word, drug trade name, original title, device manufacturer, drug manufacturer, device trade name, keyword heading word, floating subheading word, candidate term word] 88636 #5: spinal cord injury/ 57277  #6: spine injury/ 10034  #7: spinal cord transsection/ 2854  #8: spinal cord compression/ 16201  #9: 4 or 5 or 6 or 7 or 8 97037  #10: 3 and 9 261 |
| --- |

**Table 2: Basic information included in the study**

| **Author + year** | **Country** | **Type** | **Species** | **Sex** | **Weight** | **Age** | **Sample (E/C)** | **Modeling method** | **Model types** | **Exosome types** | **Exosomes sources** | **transplantation route** | **Timing (postoperative)** | **Dosage** | **Control** |
| --- | --- | --- | --- | --- | --- | --- | --- | --- | --- | --- | --- | --- | --- | --- | --- |
| Guo 2019 | Israel | Control | SD rats | Female | 200-250g | Adult | 10/15 | After laminectomy, the spinal cord was transected using a microscope at T10 level. | Transection | BMSCs-Exo | Human bone marrow mesenchymal stem cells | Intranasally） | 2-3h | 200 μg | Saline |
| Chang 2021 | China | RCT | SD rats | Male | 220-260g | Adult | 12/12 | A 10 g rod (2.5 mm in diameter) was dropped from a height of 12.5 mm onto the rats. | Contusion | BMSCs-Exo | SD rats | / | 1h | 200 μg | PBS |
| Yu 2019 | China | RCT | SD rats | Female | 230-250g | / | 20/20 | Using a standard striking device, the T10 spinal cord was hit with a striking force of 2 N. | Contusion | BMSCs-Exo | The tibia and femur of SD rats | Tail veins | 1h | 100 μg | Blank |
| Chen 2021a | China | Control | SD rats | Female | / | 8 weeks | 12/12 | Causing 50 kdyn spinal contusion injury to the rats | Contusion | NSCs-Exo | Newborn SD rats | Tail veins | 1h | 20 μg | PBS |
| Chen 2021b | China | RCT | SD rats | Male | / | 6-8 weeks | 6/6 | An aneurysm clip with the closing force of 75 g was used for compression at the T10 level for 30 s | Contusion | BMSCs-Exo | Femur of SD rats | Tail veins | 1h | 200 μg | PBS |
| Wang 2021a | China | RCT | SD rats | Female | 220-250g | / | 5/5 | The exposed spinal cord was subjected to moderate contusion injury (150 kdyn force with no dwell time) using an Infinite Horizon Impact Device. | Contusion | BMSCs-Exo | Human bone marrow mesenchymal stem cells | Subcutaneous | 1h | 100 μg | PBS |
| Fan 2021 | China | RCT | SD rats | Male | 200-250g | Adult | 6/6 | Using the New York University (NYU) Impactor to hit the exposed cord (10 g × 25 mm). | Contusion | BMSCs-Exo | / | Tail veins | Immediately | 200 μg | PBS |
| Huang 2021a | China | RCT | SD rats | Female | 250-300g | 10 weeks | 20/20 | Rats of the rest groups were hit on the T10 segment using a spinal cord impactor (PSI, IH-0400, USA), with metal rod weight of 30 g and height of 50 mm. | Contusion | BMSCs-Exo | Bone marrow of SD rats | Tail veins | Immediately | 100 μg | PBS |
| Gu 2020 | China | RCT | SD rats | Male | 220-260g | Adult | 10/10 | The rat was dropped from a height of 12.5 mm using a 10 g rod (2.5 mm in diameter). | Contusion | BMSCs-Exo | / | Tail veins | 1h | 200 μg | PBS |
| Liu 2019 | China | RCT | SD rats | Female | 170-220g | / | 10/10 | A T10 laminectomy was performed and the exposed dorsal surface of the cord was subjected to weight-drop impact using a 10g rod dropped from a height of 12.5 mm. | Contusion | BMSCs-Exo | / | Tail veins | Immediately | 200 μg | PBS |
| Luo 2020 | China | RCT | SD rats | Female | 170-220g | 12 weeks | 10/10 | A weight-drop impact was performed using a 10-g rod from a height of 12.5 mm | Contusion | BMSCs-Exo | Bone marrow of SD rats | Tail veins | Immediately | 200 μg | PBS |
| Huang 2017 | China | RCT | SD rats | / | 180-220g | / | 15/15 | Using a modified Allen’s weight drop apparatus (8-g weight at a vertical height of 40mm, 8g·40 mm) on the exposed dura of the spinal cord. | Contusion | BMSCs-Exo | Bone marrow of SD rats | Tail veins | 30min | 100 μg | PBS |
| Huang 2018 | China | RCT | SD rats | Female | 180-220g | Adult | 10/10 | Using a modified Allen’s weight drop apparatus (8 g weight from a vertical height of 40 mm, 8 g×40 mm) | Contusion | BMSCs-Exo | Bone marrow of SD rats | Tail veins | 30min | 100 μg | PBS |
| Huang 2021b | China | RCT | SD rats | / | / | / | 6/6 | The T10 segment was impacted by the spinal cord injury percussion apparatus. The weight of the metal rod reached 25 g, and the height was 50 mm. | Contusion | BMSCs-Exo | Bone marrow of SD rats | Tail veins | 1h | 100 μg | PBS |
| Wang 2021b | China | RCT | SD rats | Female | / | 6-7 weeks | 20/19 | / | / | hUC-MSC-Exo | Human cord | Tail veins | 1h | / | PBS |
| Sun 2018 | China | RCT | C57BL/6 mice | Female | 17-22g | 7-8 weeks | 8/8 | the exposed dorsal surface of the cord was subjected to a weight drop injury using a 10 g rod dropped at a height of 6.25 mm. | Contusion | hUC-MSC-Exo | Human cord | Tail veins | 30min | 200 μg | PBS |
| Zhou 2021a | China | RCT | SD rats | Female | 200-250g | 3月 | 40/40 | A T10 laminectomy was conducted, and the exposed spinal cord was subjected to a contusive wound by applying an impact of 2 N with a spinal cord impactor. | Contusion | BMSCs-Exo | The tibia of SD rats | Tail veins | 30min | 200 μg | PBS |
| Zhou 2021b | China | RCT | SD rats | Female | 200-220 | 7-8 weeks | 6/6 | Using aseptic technique, the spinal cord was exposed at the T11 vertebral level via laminectomy as previously described. This step was followed by the complete transection of the spinal cord | Transection | HpMSC-Exo | Human placentae | Tail veins | 1h | 50 μg | PBS |
| Jia 2021a | China | RCT | SD rats | Female | 230-250g | / | 10/10 | A striking device was then used to apply a 2 N striking force to the T10 spinal cord. | Contusion | BMSCs-Exo | The tibia and femur of SD rats | Tail veins | 1h | 200 μg | Saline |
| Jia 2021b | China | RCT | SD rats | Female | 230-250g | / | 10/10 | A striking device was then used to apply a 2 N striking force to the T10 spinal cord. | Contusion | BMSCs-Exo | The tibia and femur of SD rats | Tail veins | 1h | 200 μg | Saline |
| Jiang 2020 | China | RCT | C57BL/6 mice | Male | / | 6-8 weeks | 8/8 | Laminectomy was conducted to expose the spinal cord at T10 and a rod weighing 5 g was dropped from a height of 6.5 cm onto the spinal cord to induce injury using an impactor | Contusion | NSCs-Exo | / | Tail veins | Immediately | 200 μg | PBS |
| Jiang 2021 | China | RCT | SD rats | Male | 200±20g | 7 weeks | 16/16 | / | Contusion | BMSCs-Exo | Bone marrow of SD rats | Tail veins | 30min | 100 μg | PBS |
| Wang 2018 | China | RCT | SD rats | Male | 200-250g | Adult | 25/23 | A contusive injury (200 kilodyne) was applied to the exposed dura mater, using a spinal cord impactor. | Contusion | BMSCs-Exo | Femur of SD rats | Tail veins | Immediately | 200 μg | PBS |
| Kang 2020 | China | RCT | SD rats | Male | 200±20g | Adult | 8/8 | an impactor (with a diameter of 2 mm and weight of 10 g) rapidly fell from a higher place with a distance of 25 mm onto the back side of the spinal cord. | Contusion | NSCs-Exo | / | Tail veins | / | / | Saline |
| Zhao 2019 | China | RCT | Wistar rats | Male | 200-250g | Adult | 27/23 | After identifying half of the right and median veins of the spinal cord, the median vein was used as a boundary where, slightly to the left of the center, the right semicircular spinal cord was severed horizontally using an iris knife. | Hemi-sectioned | BMSCs-Exo | Femur of Wistar rats | Tail veins | 30min | 100 μg | PBS |
| Zhang 2021 | China | RCT | SD rats | Male | 200-230g | 8 weeks | 8/8 | a NYU-III weight drop apparatus (10 g weight at a vertical height of 12.5 mm, 10 g × 12.5 mm) on the exposed dura mater of the spinal cord | Contusion | BMSCs-Exo | The tibia and femur of SD rats | Tail veins | 30min | 200 μg | PBS |
| Li 2018 | China | RCT | SD rats | Male | 200-250g | Adult | 10/10 | SCI was inflicted with an aneurysm clip of 35 g closing force for 60 s at the T10 level | Contusion | BMSCs-Exo | The tibia and femur of SD rats | Tail veins | 24h | 200 μg | PBS |
| Li 2019 | China | RCT | Wistar rats | Male | 150-200g | Adult | 50/50 | A 10 g metal weight was dropped from a height of 5 cm to hit the T9– T11 spinal cord of rats in the BMSCs-Exos group and PBS group, and the metal left the spinal cord immediately after the strike | Contusion | BMSCs-Exo | Bone marrow of Wistar rats | Tail veins | Immediately | 200 μg | PBS |
| Li 2020a | China | RCT | SD rats | Male | / | / | 10/10 | The T10 spinous process was subjected to impact trauma by compression at an interval of 12.5 mm for 20 s to produce severe injury. | Contusion | BMSCs-Exo | The tibia and femur of SD rats | Tail veins | 24h | 100 μg | Saline |
| Li 2020b | China | RCT | SD rats | Female | 220-250g | / | 8/8 | A lesion gap of 4.0 ± 0.5 mm was made by complete transection of the T9-T10 spinal cord segment | Transection | BMSCs-Exo | Human bone marrow mesenchymal stem cells | Tail veins | Immediately | 100 μg | PBS |
| Liu 2020 | China | RCT | C57BL/6 mice | Male | / | 6-8 weeks | 8/8 | A spinal cord impactor was used to create injury by dropping a rod (weighing 5 g) onto the spinal cord from a height of 6.5 cm. | Contusion | BMSCs-Exo | / | Tail veins | Immediately | 200 μg | PBS |
| Liu 2021 | China | RCT | SD rats | Male | 250-300g | 6-8weeks | 6/6 | SCI was inflicted by dropping a 10 g rod from a height of 6.5 cm onto the spinal cord. | Contusion | BMSCs-Exo | The tibia and femur of SD rats | Tail veins | Immediately | 100 μg | PBS |

**Table 3 Quality of evidence in included systematic reviews with GRADE**

| **Outcomes** | **Number of studies** | **Risk of bias** | **Inconsistency** | **Indirectness** | **Imprecision** | **Publication bias** | **Quality of evidence** |
| --- | --- | --- | --- | --- | --- | --- | --- |
| BBB score | 29 | Serious^a^ | Serious^b^ | Not serious | Serious^c^ | Serious^d^ | Very low |
| Bax | 7 | Serious^a^ | Not serious | Not serious | Serious^c^ | Serious^d^ | Low |
| Bcl-2 | 8 | Serious^a^ | Not serious | Not serious | Serious^c^ | Serious^d^ | Low |
| IL-1β | 7 | Serious^a^ | Not serious | Not serious | Serious^c^ | Serious^d^ | Low |
| IL-6 | 4 | Serious^a^ | Not serious | Not serious | Serious^c^ | Serious^d^ | Low |
| TNF-α | 7 | Serious^a^ | Not serious | Not serious | Serious^c^ | Serious^d^ | Low |
| IL-4 | 3 | Serious^a^ | Not serious | Not serious | Serious^c^ | Serious^d^ | Low |
| IL-10 | 3 | Serious^a^ | Not serious | Not serious | Serious^c^ | Serious^d^ | Low |

a: The design of the experiment with a large bias in random, distributive hiding or blind; b: The confidence interval overlaps less and the I2 is larger; c: The confidence interval is too wide or contains invalid values; d: Fewer studies are included and there may be greater publication bias.
